# Alzheimer's disease-associated peptide Aβ_42_ mobilizes ER Ca^2+^ via InsP_3_R-dependent and -independent mechanisms

**DOI:** 10.3389/fnmol.2013.00036

**Published:** 2013-11-05

**Authors:** Laura E. Jensen, Geert Bultynck, Tomas Luyten, Hozeefa Amijee, Martin D. Bootman, H. Llewelyn Roderick

**Affiliations:** ^1^Babraham Institute, Babraham Research CampusBabraham, Cambridge, UK; ^2^Laboratory of Molecular and Cellular Signaling, Department Molecular Cell Biology, K.U. LeuvenLeuven, Belgium; ^3^Senexis, Babraham Research CampusBabraham, Cambridge, UK

**Keywords:** Alzheimer's disease, Aβ oligomers, calcium/Ca^2+^, InsP_3_/IP_3_, InsP_3_ receptors/InsP_3_Rs, endoplasmic reticulum/ER

## Abstract

Dysregulation of Ca^2+^ homeostasis is considered to contribute to the toxic action of the Alzheimer's disease (AD)-associated amyloid-β-peptide (Aβ). Ca^2+^ fluxes across the plasma membrane and release from intracellular stores have both been reported to underlie the Ca^2+^ fluxes induced by Aβ_42_. Here, we investigated the contribution of Ca^2+^ release from the endoplasmic reticulum (ER) to the effects of Aβ_42_ upon Ca^2+^ homeostasis and the mechanism by which Aβ_42_ elicited these effects. Consistent with previous reports, application of soluble oligomeric forms of Aβ_42_ induced an elevation in intracellular Ca^2+^. The Aβ_42_-stimulated Ca^2+^ signals persisted in the absence of extracellular Ca^2+^ indicating a significant contribution of Ca^2+^ release from the ER Ca^2+^ store to the generation of these signals. Moreover, inositol 1,4,5-trisphosphate (InsP_3_) signaling contributed to Aβ_42_-stimulated Ca^2+^ release. The Ca^2+^ mobilizing effect of Aβ_42_ was also observed when applied to permeabilized cells deficient in InsP_3_ receptors, revealing an additional direct effect of Aβ_42_ upon the ER, and a mechanism for induction of toxicity by intracellular Aβ_42_.

## Introduction

Alzheimer's disease (AD) is a progressive and irreversible brain disorder, which results in severe memory loss, behavioral as well as personality changes and a decline in cognitive abilities. While the most common type of AD remains idiopathic in origin, with age the most significant risk factor for disease onset (sporadic AD, sAD), ~5% of cases show a Mendelian pattern of inheritance (familial AD, fAD). The amyloid β-peptide (Aβ) is hypothesized to be central to the pathogenesis of both sporadic and familial AD (Hardy and Selkoe, 2002). Aβ is a small, hydrophobic polypeptide, consisting of 39–42 amino acid residues, which occurs principally as a 40 or 42 amino acid peptide, Aβ_40_ and Aβ_42_, respectively (Zhang et al., 2011). An imbalance between the production and clearance of Aβ, as occurs in fAD and sAD, respectively, leads to the accumulation of Aβ and, in turn, to its aggregation. This aggregation process represents a critical step in the pathogenesis of AD because the neurotoxic properties of Aβ are associated only with aggregated forms of the peptide (Kuperstein et al., 2010). Protein aggregation is highly dynamic and involves a wide range of intermediate structures such as oligomers, comprising dimers, trimers, dodecamers, and higher-molecular weight complexes, before aggregating into protofibrils and finally into mature amyloid fibrils (Dobson, 2003).

A mounting body of evidence now suggests that soluble oligomeric forms of Aβ constitute the primary neurotoxic species rather than monomers or fibrils (Lambert et al., 1998; Chromy et al., 2003; Gong et al., 2003; Demuro et al., 2005; Klyubin et al., 2005). Soluble oligomers have proved toxic when applied to cultured cells and primary neuronal cultures *in vitro* (Lambert et al., 1998; Bucciantini et al., 2002; Dahlgren et al., 2002; Kayed et al., 2003; Whalen et al., 2005). In addition, they are capable of inducing cognitive deficits when administered *in vivo* (Cleary et al., 2005; Rowan et al., 2007) and adversely affect hippocampal LTP *in vivo* (Walsh et al., 2002; Cleary et al., 2005; Klyubin et al., 2009, 2011).

Dysregulation of intracellular Ca^2+^ homeostasis is associated with cell exposure to Aβ and likely underlies its neurotoxic effects (Bezprozvanny and Mattson, 2008; Green and Laferla, 2008; Berridge, 2010; Demuro et al., 2010). A number of mechanisms by which Aβ elicits its effects on intracellular Ca^2+^ homeostasis have been put forward. These include direct effects on the plasma membrane, where it has been proposed to destabilize its structure (Mueller et al., 1995; Mason et al., 1996), to induce a generalized increase in membrane permeability (Bucciantini et al., 2002; Kayed et al., 2003) or to insert into the membrane forming cation-conducting pores (Arispe et al., 1993; Mueller et al., 1995; Mason et al., 1996; Bucciantini et al., 2002; Kayed et al., 2003; Kawahara, 2004; Simakova and Arispe, 2006; Arispe et al., 2007; Demuro et al., 2011). Aβ has also been reported to activate plasma membrane receptors, including *N*-methyl-d-aspartate (NMDA) receptors coupled to Ca^2+^ influx (Guo et al., 1996; Dobson, 2003; Blanchard et al., 2004; De Felice et al., 2007), to alter neuronal excitability which, in turn, influences the extent of Ca^2+^ influx (Good et al., 1996) and to induce dysregulation of endoplasmic reticulum (ER) Ca^2+^ homeostasis (Ferreiro et al., 2004, 2006; Resende et al., 2008). In addition to acting from the extracellular space, where it accumulates in the diseased brain, Aβ also has an intracellular site of action (Wirths et al., 2004). Indeed, as a result of uptake from the extracellular space or via its intracellular synthesis and processing, Aβ has been reported to accumulate within the cell (Pierrot et al., 2004; Bayer and Wirths, 2011; Kaminski Schierle et al., 2011). This intracellular Aβ is also neurotoxic and has been shown to target the ER and the mitochondria, inducing a stress response and causing permeability transition, respectively (Yao et al., 2009; Umeda et al., 2011).

In this study, we investigated (1) the contribution of Ca^2+^ mobilization from the ER to the increase in intracellular Ca^2+^ induced by oligomeric Aβ_42_, (2) the mechanism (s) by which Aβ_42_ elicited this effect, (3) the capacity for Aβ_42_ to mobilize Ca^2+^ directly from the ER. To allow isolation of effects on the ER from other plasma membrane targets of Aβ_42_, model cells systems were used that allowed fundamental aspects of ER Ca^2+^ regulation to be studied. We determined that Ca^2+^ release from the intracellular ER substantially contributed to the increase in intracellular Ca^2+^ concentration induced by oligomeric Aβ_42_. The Aβ_42_-induced Ca^2+^ elevation comprised InsP_3_ dependent and independent components. Using DT40 cells deficient in the three InsP_3_R isoforms that were permeabilized to allow direct access of Aβ_42_ to the ER, we also demonstrated that it had the capacity to release Ca^2+^ from the ER independent of InsP_3_Rs. Together, these data place the ER and Ca^2+^ released from it as central to the actions of both extracellular Aβ and Aβ that has reached an intracellular location.

## Materials and methods

### Materials

Peptides were purchased from The American Peptide Company and rPeptide. Cell culture reagents and chemicals were from Invitrogen or Sigma, unless otherwise stated.

### Cell culture

Human neuroblastoma SH-SY5Y cells were cultured in F-12 Nutrient Mixture (Ham) containing FBS (10%), penicillin (100 units/ml), streptomycin (100 μg/ml), non-essential amino acids (0.1 mM), and L-glutamine (2 mM). Prior to all experiments, SH-SY5Y cells were cultured overnight in Opti-MEM Reduced Serum Medium, containing FBS (1.5%), penicillin (100 units/ml), streptomycin (1.0 μg/ml), non-essential amino acids (0.1 mM), and L-glutamine (2 mM). For live-cell Ca^2+^ imaging experiments, cells were plated onto poly-L-lysine-coated coverslips at a density of 3.2 × 10^4^ cell/cm^2^. For the MTT reduction assay, cells were plated at a density of 9 × 10^3^ cells/cm^2^. To overexpress GFP-tagged type 1 InsP_3_ 5′-Phosphatase (GFP-5′P) or GFP (Peppiatt et al., 2004; Higazi et al., 2009), cells were infected with adenovirus for 8 h prior to overnight culture. Culture of DT40 cells and DT40 cells deficient in the three InsP_3_R isoforms (DT40 TKO) was performed as previously described (Tovey et al., 2006).

### Preparation of Aβ_42_ oligomers

Wild type and scrambled Aβ_42_ were obtained at a purity of >95%. Peptide mass was verified by matrix-assisted laser desorption/ionization time-of-flight mass spectrometry and peptides from the same batch were used throughout. Samples of synthetic Aβ_42_ oligomers were prepared as previously described (Demuro et al., 2005) and remained stable for at least 3 weeks. Samples of Aβ_1–42_ scrambled peptide (KVKGLIDGAHIGDLVYEFMDSNSAIFREGVGAGHVHVAQVEF) were prepared in the same way as Aβ_42_ oligomers. All Aβ samples were stored at 4°C and were used within 10–15 days of preparation. Toxicity of Aβ_42_ preparations was confirmed by MTT assay before use in Ca^2+^ imaging experiments (Figure S1A). The oligomeric nature of the Aβ_42_ preparation was established by surface plasmon resonance (SPR) spectroscopy using an antibody specific to oligomeric Aβ_42_ (Figure S1B). All Aβ_42_ concentrations stated are based on the molar mass of the peptide.

### Live cell Ca^2+^ imaging

Methods for single cell analysis of intracellular Ca^2+^ concentration were as previously described (Peppiatt et al., 2003). Cells were loaded at 37°C with 2 μM of the acetoxymethyl (AM) ester form of fura-2 for 30 min followed by an equivalent period in dye free media to allow de-esterification of the indicator. Imaging experiments were performed using either Ca^2+^-containing (121 mM NaCl, 5.4 mM KCl, 0.8 mM MgCl_2_, 1.8 mM CaCl_2_, 6 mM NaHCO_3_, 25 mM HEPES, 5.5 mM glucose, pH 7.3) or Ca^2+^ free (as for Ca^2+^ containing with 1.8 mM CaCl_2_ replaced with 1 mM EGTA) buffer as indicated. Fura-2 imaging was carried out using an imaging system configured around a Nikon TE300 inverted epi-fluorescence microscope equipped with a 20× 0.75 NA multi-immersion objective. Samples were illuminated by alternate excitation at 340 and 380 nm using a Sutter filter changer (340HT15 and 380HT15; Sutter Industries) and emitted light was filtered at >460 nm (1 ratio pair per 2 s). Images were captured using a Hamamatsu ORCA ER CCD camera. The imaging system was controlled with Ultraview software (PerkinElmer Life Sciences Ltd., UK). Acquired images were processed with Ultraview software and analyzed in MATLAB. Background subtracted fura-2 ratios were calibrated according to standard procedures (Grynkiewicz et al., 1985), using the maximum and minimum ratio values obtained through exposing cells sequentially to Ca^2+^ free and Ca^2+^ containing imaging buffer to which 2 μM ionomycin had been added. Parameters analyzed from the Ca^2+^ responses included the peak amplitude, the time to peak and the integral of the response (the area under the curve) and the percentage of responding cells.

InsP_3_-induced Ca^2+^ release (IICR) from permeabilized wild type and InsP_3_R null DT40 cells (three InsP_3_R isoforms deleted by homologous recombination; DT40 TKO) (Sugawara et al., 1997) was performed as previously described (Tovey et al., 2006). Briefly, the ER of cells was loaded with the low-affinity Ca^2+^ indicator mag-fluo-4 and Aβ-induced Ca^2+^ release was measured from the saponin-permeabilized cells using a fluorescence plate reader (FlexStation 3, Molecular Devices).

### MTT reduction assay

The Cell Titer 96 Non-Radioactive Cell Proliferation Assay (Promega) was used to validate the cytotoxic effect of Aβ_42_ on SH-SY5Y cells and was performed according to manufacturer's instructions. Briefly, cells were incubated with Aβ_42_ (*n* = 4) for 24 h prior to the addition of the MTT dye solution and a further 4 h incubation at 37°C, 5% CO_2_. Thereafter, the solubilization/stop solution was added and incubated overnight at room temperature. Absorbances were read at 570 nm with a reference wavelength of 650 nm using a fluorescence plate reader (Synergy HT, BIO-TEK). The data is expressed as the percentage of MTT reduction relative to both live- and dead-cell controls and thus represents the percentage of viable cells. Aβ_42_ samples were considered to be toxic if 25–40% of cells remained metabolically healthy at an Aβ_42_ concentration of 1 μ M and if more than 50% remained metabolically healthy at a concentration of 100 nM.

### Statistical analysis

Data is presented as the mean value of the combined datasets ± SEM. Statistical significance was determined by Student's *t*-test (two-tailed). Data was accepted as significant when *p* < 0.05 and is denoted by ^*^p < 0.05, ^**^p < 0.01, or ^***^p < 0.001.

## Results

### Intracellular Ca^2+^ is elevated in cells exposed to oligomeric Aβ_42_

**Figure 1:**
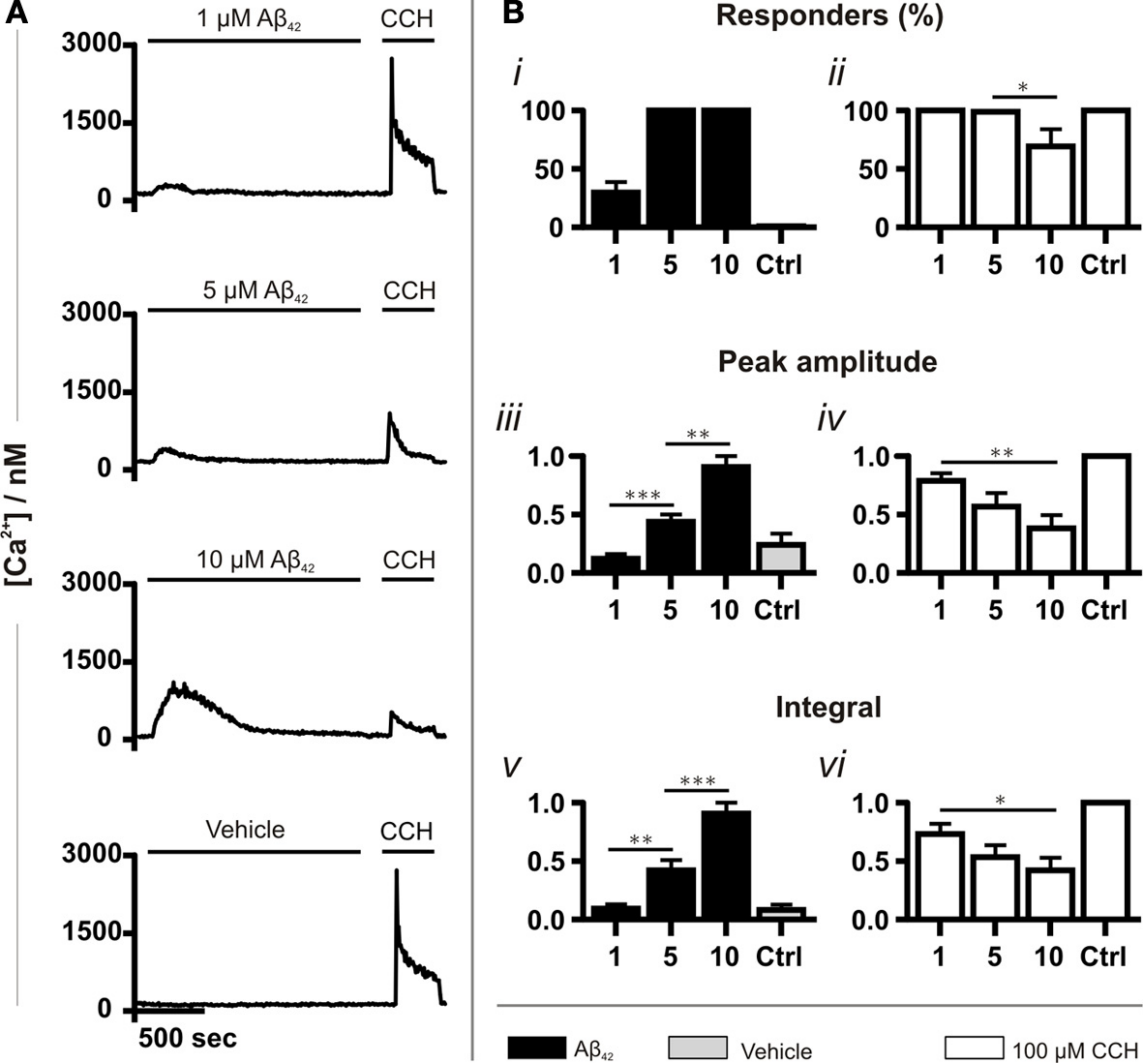
**Aβ_42_ oligomers induce Ca^2+^ transients in a concentration-dependent manner. (A)** Example fura-2 Ca^2+^ traces of SH-SY5Y cells exposed to a concentration range of Aβ_42_ oligomers followed by 100 μM CCH. A trace taken from cells in which Aβ_42_ oligomers were substituted with double-distilled water (dd H_2_O; vehicle) is also shown (for each group, *n* > 744 cells). **(B)** Quantitative analysis of the Ca^2+^ responses illustrated in A. The magnitude of Ca^2+^ responses elicited by Aβ_42_ oligomers, dd H_2_O and CCH is presented as **(Bi,ii)** percentage of responding cells, **(Biii,iv)** peak amplitude and **(Bv,vi)** integral of the response. Aβ_42_ oligomer-induced Ca^2+^ transients were normalized to the responses induced with the highest concentration (10 μM) of the respective Aβ_42_ preparation. CCH-induced Ca^2+^ responses were normalized to control experiments conducted on the same experimental day. Bar graphs are mean ± SEM from at least three independent experiments. ^*^p < 0.05; ^**^p < 0.01; ^***^p < 0.001.

Experiments were first performed to validate the Ca^2+^ mobilizing properties of oligomeric Aβ_42_ over the concentration range of its toxicity. Application of Aβ_42_ spanning its cytotoxic range (1, 5 and 10 μM) caused an elevation in intracellular Ca^2+^ (Figure 1A). The increase in cytosolic Ca^2+^ concentration immediately followed the addition of Aβ_42_, developed to a peak within minutes of application and subsequently returned to baseline, despite the continued presence of the peptide. No Ca^2+^ responses were detected when Aβ_42_ below 1 μM was applied (data not shown). Between 1 μM and 10 μM Aβ, the number of responding cells, the peak amplitude and the integral of the Ca^2+^ responses increased in a concentration-dependent manner. The number of responding cells reached 100% at 5 μM Aβ_42_ (Figures 1Bi,iii,v). To test cell viability as well as to determine whether metabotropic Ca^2+^ signaling was affected by Aβ, carbachol (CCH) was applied subsequent to Aβ. CCH elicited Ca^2+^ responses in 100% of cells pre-exposed to 1 or 5 μM oligomeric Aβ_42_ or to a vehicle control (10%) (Figures 1Bii,iv,vi). At 10 μM Aβ, however, the number of cells responding to CCH was significantly reduced (Figure 1Bii). The peak amplitude and integral of the Ca^2+^ responses to CCH subsequently applied were inversely related to the magnitude of the Ca^2+^ responses elicited by oligomeric Aβ_42_ (Figures 1Biv,vi). This observation suggested that exposure to Aβ_42_ oligomers was depleting the intracellular CCH-sensitive ER Ca^2+^ store. These Ca^2+^ mobilizing effects of oligomeric Aβ_42_ were significantly greater than observed in cells exposed to Aβ_42_ that had been prepared in a manner to yield a monomeric form of the peptide (Figure S2), S1B). From these results, due to its potency in mobilizing Ca^2+^ whilst preserving agonist responses, a concentration of 5 μM oligomeric Aβ_42_ was selected for use in subsequent experiments.

### Aβ_42_ oligomer-induced Ca^2+^ transients are peptide sequence specific

**Figure 2:**
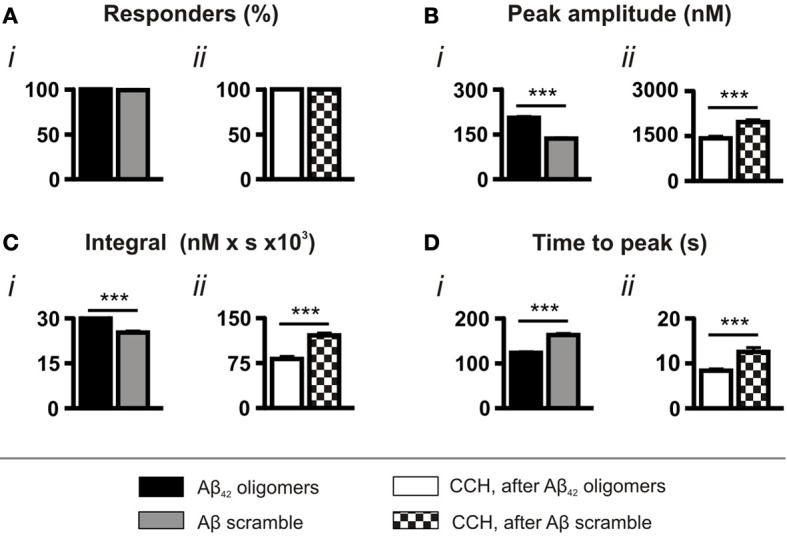
**Aβ_42_ oligomer-induced Ca^2+^ transients are sequence specific.** Bar charts illustrating the magnitude of Ca^2+^ responses elicited by SH-SY5Y cells following the application of 5 μM Aβ_42_ oligomers or Aβ scramble and 100 μM CCH (*n* > 370 cells). Data is presented as **(A)** percentage of responding cells, **(B)** peak amplitude, **(C)** integral of the response, **(D)** time to peak. Bar graphs are mean ± SEM from at least three independent experiments. ^***^p < 0.001.

As a control for the application of peptide, experiments were also performed using a scrambled Aβ sequence, which had been prepared in the same manner as the wild type Aβ_42_. Although significantly less toxic than the wild type sequence (Figure S1A), scrambled Aβ peptide also evoked Ca^2+^ responses in all cells (Figure 2Ai). However, consistent with its lower toxicity, both the amplitude and the integral of the Ca^2+^ transients elicited by scrambled Aβ were significantly lower than those induced by oligomeric Aβ_42_ and, in addition, they required a significantly longer time to reach peak (Figures 2Bi,Ci,Di). Furthermore, concordant with the less potent effect of scrambled Aβ in mobilizing intracellular Ca^2+^, the amplitude and integral of CCH-induced Ca^2+^ transients elicited following prior exposure to scrambled Aβ were significantly greater than those stimulated following prior exposure to oligomeric Aβ_42_ (Figures 2Bii,Cii,Dii).

Taken together, the comparison of the effects of Aβ scramble and oligomeric Aβ_42_ demonstrates that the amino acid sequence of Aβ_42_ has potent Ca^2+^ mobilizing properties, which are distinct from the action of Aβ scramble.

### Aβ_42_ oligomers mobilize Ca^2+^ from intracellular stores

The reduced magnitude of CCH-induced Ca^2+^ signals observed in cells previously exposed to oligomeric Aβ_42_ suggested that this form of Aβ_42_ was exerting an effect on intracellular Ca^2+^ stores. Therefore, we tested the relative contributions of Ca^2+^ influx from the extracellular space and its release from intracellular stores to Aβ_42_-induced Ca^2+^ transients.

**Figure 3:**
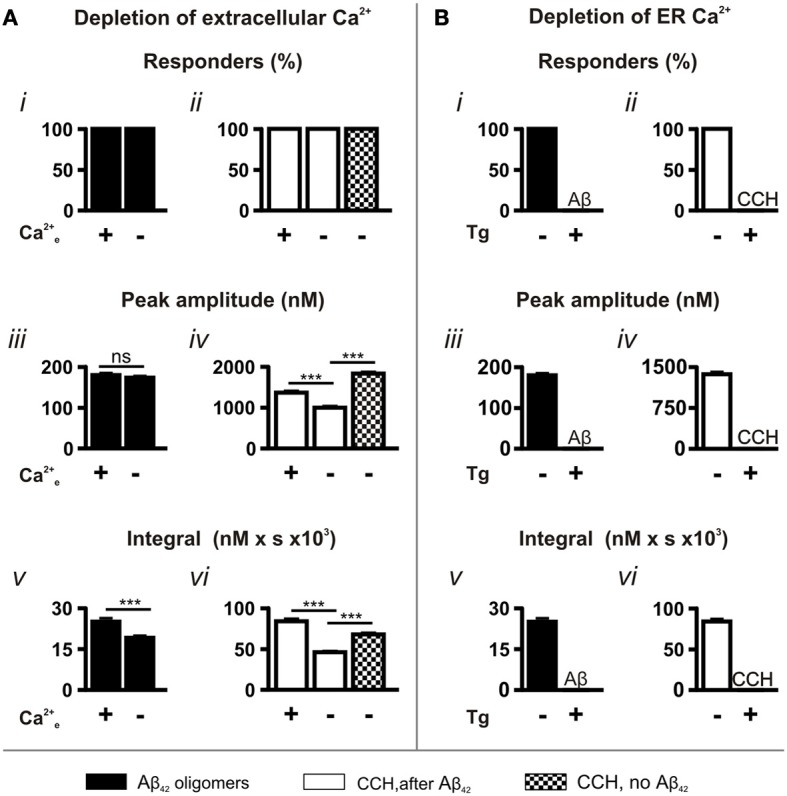
**Aβ_42_ oligomer-induced Ca^2+^ transients arise through release from the ER Ca^2+^ store.** Bar charts illustrating the magnitude of Ca^2+^ responses elicited following the manipulation of **(A)** extracellular (*n* > 218 cells) and **(B)** ER Ca^2+^ concentrations (*n* > 512 cells). The magnitude of Ca^2+^ responses elicited by 5 μM Aβ_42_ oligomers and 100 μM CCH is presented as **(i,ii)** percentage of responding cells, **(iii,iv)** peak amplitude and **(v,vi)** integral of the response. Bar graphs are mean ± SEM from at least three independent experiments. ^***^P < 0.001.

To determine the contribution of extracellular Ca^2+^ and Ca^2+^ influx to Aβ_42_ oligomer-induced Ca^2+^ transients, we performed experiments using Ca^2+^-free imaging buffer. Under these conditions, Aβ_42_ oligomers retained their ability to induce Ca^2+^ responses, with 100% of cells responding (Figure 3Ai). While no significant difference was observed in the peak amplitude (Figure 3Aiii) of Aβ_42_ oligomer-induced Ca^2+^ transients, the integral of the response was significantly decreased in the absence of extracellular Ca^2+^ (Figure 3Av).

In contrast to the Aβ_42_ oligomer-induced Ca^2+^ response, the peak amplitude and the integral of the Ca^2+^ responses to CCH applied following Aβ_42_ oligomer exposure were significantly decreased by removal of extracellular Ca^2+^ from the imaging buffer (CCH, after Aβ_42_; Figures 3Aiv,vi). This effect on the CCH-induced Ca^2+^ responses is likely due to lack of store-operated Ca^2+^ entry, which would replenish the Ca^2+^ released from stores by Aβ_42_. Indeed, the peak amplitude and the integral of CCH-induced Ca^2+^ responses elicited in Ca^2+^ free buffer were significantly greater in naïve cells (CCH, no Aβ_42_) than when Aβ_42_ oligomers were previously applied (Figures 3Aiv,vi). Since Aβ_42_ oligomer-induced Ca^2+^ transients were not significantly affected by removal of extracellular Ca^2+^, these results suggest that oligomeric Aβ_42_ and CCH mobilize Ca^2+^ from a common intracellular Ca^2+^ pool.

The requirement of Ca^2+^ release from the ER Ca^2+^ store for the Ca^2+^ transients elicited by Aβ-induced was next investigated. To this end, ER Ca^2+^ stores were depleted by exposure of cells the SERCA pump inhibitor thapsigargin (Tg; 2 μM, 15 min) prior to the application of Aβ_42_. In the absence of replete ER Ca^2+^ stores, Aβ_42_-induced Ca^2+^ transients were completely abrogated (Figures 3Bi,iii,v). Similarly, CCH-induced Ca^2+^ responses were eliminated in Tg-treated cells (Figures 3Bii,Biv,Bvi), confirming the effect of Tg. Taken together, these experiments establish that Aβ_42_ oligomers mobilize Ca^2+^ from the ER.

### Aβ_42_-induced Ca^2+^ release occurs in part through InsP_3_Rs

Having determined that Aβ_42_ oligomers mobilize Ca^2+^ from the intracellular ER Ca^2+^ store, we aimed to identify the mechanism by which Ca^2+^ release occurs. We therefore tested whether Aβ_42_ was causing Ca^2+^ release from the ER through activation of InsP_3_R or ryanodine receptor (RyR) Ca^2+^ release channels localized to this organelle.

Although SH-SY5Y cells have been reported to express functional RyRs, application of caffeine (10 mM), an agonist of the three RyR isoforms (10 mM) did not elicit a Ca^2+^ response in the SH-SY5Y cells used in this study (Figure S2A). Furthermore, the neuronally-expressed type 2 RyR could not be detected by immunoblot analysis (Figure S2B). Based on these observations, a role for RyR2 in Aβ_42_ oligomer-mediated Ca^2+^ release was ruled out.

SH-SY5Y cells express InsP_3_Rs and elicit robust Ca^2+^ responses to InsP_3_-generating agonists including CCH (Tovey et al., 2001) (Figures 1–3). Therefore, we focused our investigation on the contribution of InsP_3_Rs to Aβ_42_-induced Ca^2+^ transients. To abrogate InsP_3_-mediated Ca^2+^ responses, InsP_3_ signaling was inhibited pharmacologically with 10 mM caffeine (Parker and Ivorra, 1991; Bezprozvanny et al., 1994) or was prevented by adenoviral-mediated overexpression of GFP-5′P, which metabolizes the second messenger InsP_3_ to inactive InsP_2_ (Higazi et al., 2009). To exclude the contribution of Ca^2+^ influx to the Aβ_42_ oligomer-induced Ca^2+^ transients, these experiments were performed in the absence of extracellular Ca^2+^.

**Figure 4:**
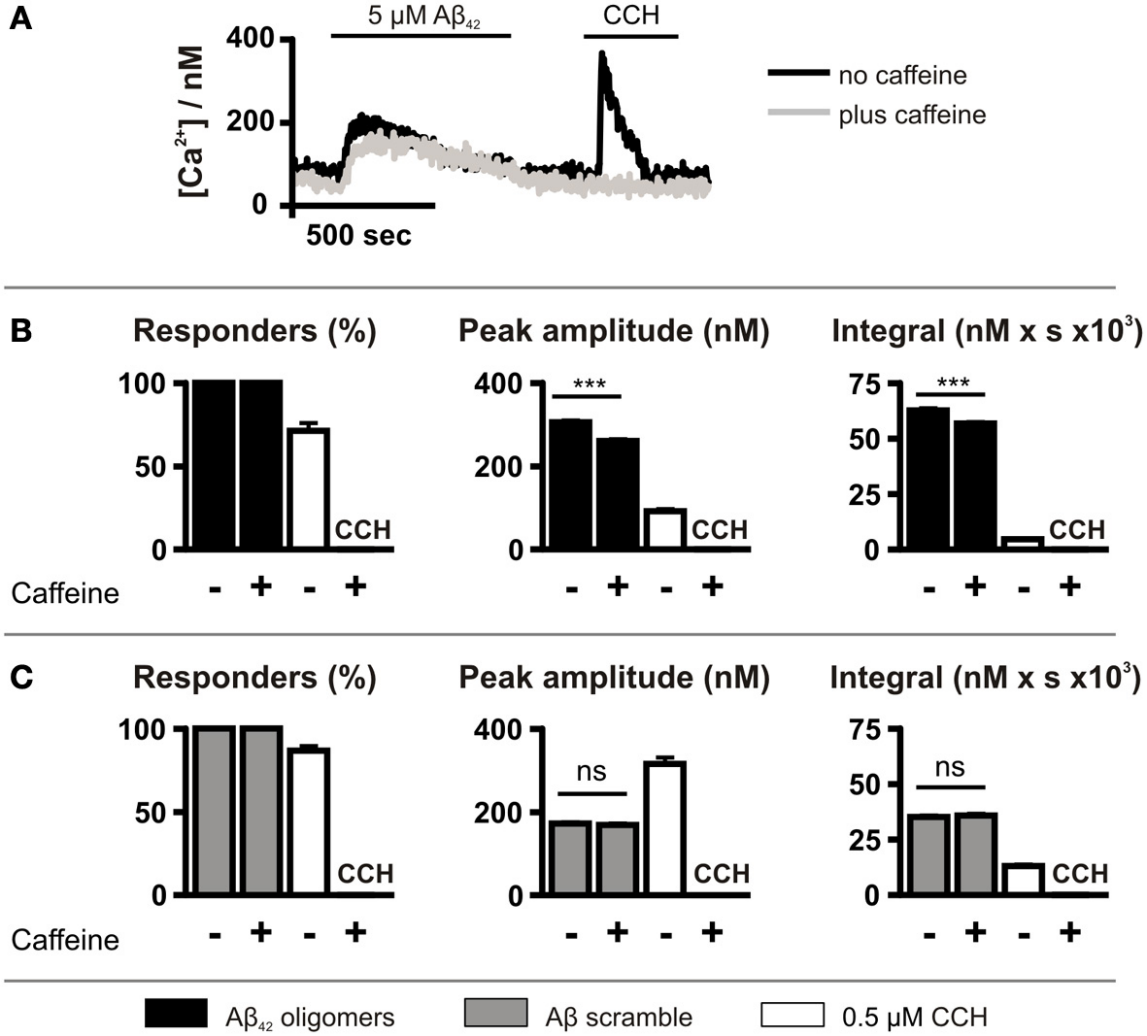
**Aβ_42_ oligomer-induced Ca^2+^ release is sensitive to caffeine. (A)** Imaging protocol employed to investigate the involvement of InsP_3_Rs in Aβ_42_ oligomer-mediated Ca^2+^ release from the ER. InsP_3_Rs were inhibited by co-administration of caffeine. **(B,C)** Bar charts illustrating the magnitude of Ca^2+^ responses elicited by SH-SY5Y cells following the application of 5 μM Aβ_42_ oligomers (*n* > 780 cells) **(B)** or Aβ scramble (*n* > 144 cells) **(C)** and 0.5 μM CCH (*n* = 512 cells) in the presence or absence of 10 mM caffeine. Data is presented as percentage of responding cells, peak amplitude and integral of the response. Bar graphs are mean ± SEM from at least three independent experiments. ^***^P < 0.001.

Caffeine application did not affect the number of cells exhibiting Ca^2+^ responses following Aβ_42_ oligomer application, with 100% of cells responding (Figure 4B). However, caffeine significantly decreased the peak amplitude and the integral of the Aβ_42_ oligomer-induced Ca^2+^ transients (Figure 4B). In contrast, Aβ scramble-induced Ca^2+^ transients were unaffected by caffeine application (Figure 4C). Ca^2+^ responses to 0.5 μM CCH were abolished by caffeine, demonstrating its inhibitory effect upon IICR (Figures 4A–C).

**Figure 5:**
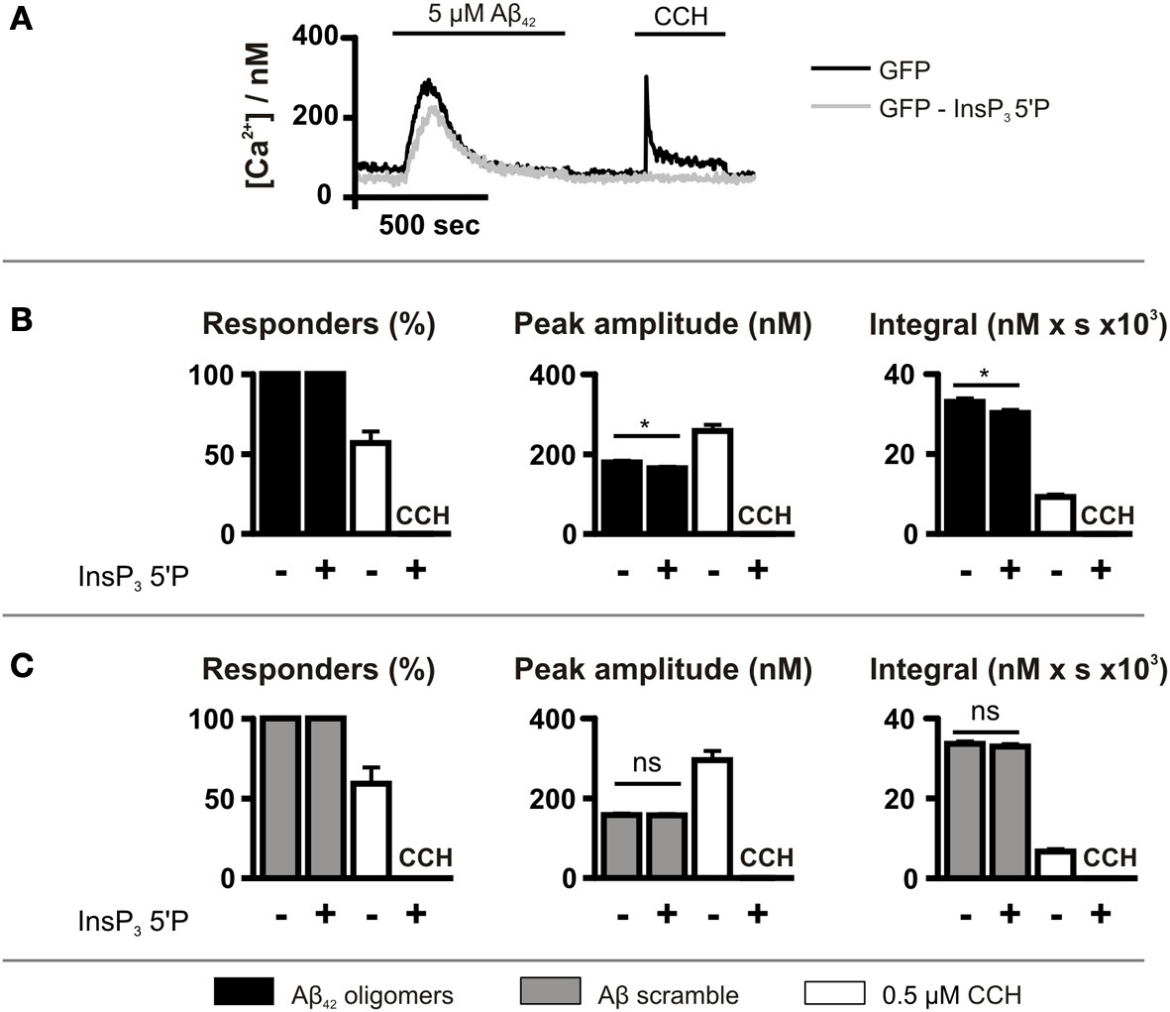
**Aβ_42_ oligomer-induced Ca^2+^ release occurs is reduced by InsP_3_ 5′P expression. (A)** Imaging protocol employed to investigate the involvement of InsP_3_Rs in Aβ_42_ oligomer-mediated Ca^2+^ release from the ER. InsP_3_ was metabolized by overexpression of InsP_3_ 5′P. **(B,C)** Bar charts illustrating the magnitude of Ca^2+^ responses elicited by SH-SY5Y cells infected with InsP_3_ 5′P or GFP alone following the application of 5 μM Aβ_42_ oligomers (*n* > 207 cells) **(B)** or Aβ scramble (*n* > 115 cells) **(C)** and 0.5 μM CCH (*n* > 55 cells). Data is presented as percentage of responding cells, peak amplitude and integral of the response. Bar graphs are mean ± SEM from at least three independent experiments. ^*^P < 0.05.

Although caffeine inhibits InsP_3_Rs (Bezprozvanny et al., 1994), it also acts on targets other than the InsP_3_R such as cyclic nucleotide phosphodiesterases and phospholipase C (PLC) (Toescu et al., 1992; Taylor and Broad, 1998). Therefore, to investigate further the role of InsP_3_ signaling in the generation of Aβ_42_ oligomer-induced Ca^2+^ transients, InsP_3_ signaling was inhibited by GFP-5′P overexpression. Using this strategy, InsP_3_-mediated Ca^2+^ signals induced by CCH were prevented, validating this approach for suppression of InsP_3_ signaling (Figure 5A). As observed for caffeine, however, GFP-5′P overexpression did not prevent Aβ_42_ oligomer-induced Ca^2+^ transients, with 100% of cells responding (Figure 5B). However, the peak amplitude and the integral of Aβ_42_ oligomer-induced Ca^2+^ transients were significantly decreased by overexpression of GFP-5′P (Figure 5B) when compared to the magnitude of Ca^2+^ transients in control cells, expressing GFP alone. Significantly, Aβ scramble-induced Ca^2+^ transients were not affected by GFP-5′P overexpression with no significant impact of its expression upon the peak amplitude or the integral of Aβ scramble-induced Ca^2+^ transients (Figure 5C). Taken together, these results demonstrate that Ca^2+^ transients elicited by Aβ_42_ oligomers arise as a result of release from the ER intracellular Ca^2+^ store and that activation of InsP_3_Rs contributes to this effect.

### Aβ_42_ oligomer-induced Ca^2+^ leak from the ER

The data presented above indicates that externally applied Aβ_42_ rapidly induces an increase on cytosolic Ca^2+^ that involves InsP_3_-dependent and -independent Ca^2+^ release from the ER. Since Aβ_42_ has also been shown to elicit some of its cytotoxic effects as a result of intracellular accumulation (Wirths et al., 2004), we investigated whether it mobilized Ca^2+^ from the ER when directly applied. We also tested whether InsP_3_Rs were required for its intracellular action.

**Figure 6:**
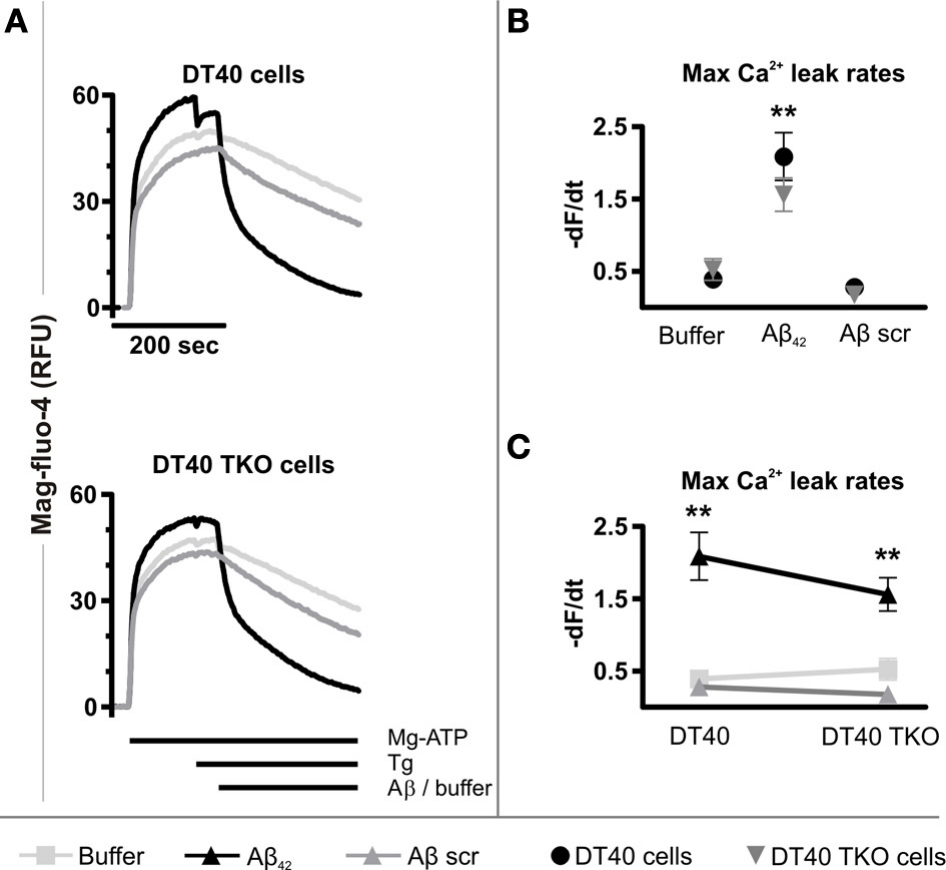
**Aβ_42_ oligomers stimulate Ca^2+^ release from the ER of permeabilized cells. (A)** Representative traces of mag-fluo-4 fluorescence (relative fluorescence units, RFU) in permeabilized DT40 cells and DT40 TKO cells, illustrating changes in ER luminal Ca^2+^ levels as a function of time in response to Aβ_42_ oligomers, Aβ scramble and buffer alone. Data represents the mean of three measurements. **(B,C)** Initial quantitative analysis of the maximum Ca^2+^ leak rates from the ER of permeabilized DT40 cells and DT40 TKO cells triggered by Aβ_42_ oligomers, Aβ scramble and buffer, respectively. The maximal Ca^2+^ leak rate was calculated by taking the maximal value of the first derivative of the fluorescence values to the time. Data were calculated as −dF/dt and represents the mean Ca^2+^ leak rate obtained from three measurements in RFU over time (RFU/s). ^**^p < 0.01.

To this end, an established permeabilized cell high-throughput functional assay of ER Ca^2+^ release was used (Tovey et al., 2006). This model uses as substrate for specific analysis of ER Ca^2+^ release, a plasma membrane-permeabilized preparation of the DT40 chicken B-lymphocyte cell line. A derivative of this cell line in which the 3 InsP_3_R isoforms have been deleted by homologous recombination (DT40 TKO), allows the requirement for InsP_3_Rs for Ca^2+^ release to be tested (Sugawara et al., 1997). Cell permeabilization and substantial dilution in intracellular buffer rules out the contribution of endogenously generated InsP_3_ to signaling in this assay. Using this assay, a significantly greater InsP_3_ independent ER Ca^2+^ leak was observed in both wild-type (*p* = 0.002) and DT40 TKO cells (*p* = 0.0195) exposed to Aβ_42_ oligomers compared to the passive Ca^2+^ leak detected in each cell type (Figures 6A,B). The maximal Ca^2+^ leak rate induced by Aβ_42_ oligomers was not significantly different between wild-type and DT40 TKO cells (*p* = 0.2606, Figure 6C), suggesting that InsP_3_Rs were not required for Aβ_42_ oligomers to trigger Ca^2+^ release.

Aβ scramble did not increase the rate of the Ca^2+^ leak in DT40 cells (*p* = 0.2528) or in DT40 TKO cells (*p* = 0.0993) compared to the passive Ca^2+^ leak observed in each cell type (Figure 6B), and thus there was no significant difference in the maximal Ca^2+^ leak rate following Aβ scramble application between these two cell types (*p* = 0.2522, Figure 6C). Importantly, a significant difference between the Ca^2+^ leak rates triggered by exposure to Aβ_42_ oligomers and Aβ scramble in wild-type DT40 cells (*p* = 0.0056) and DT40 TKO cells (*p* = 0.0045) was observed, indicating that Aβ-induced Ca^2+^ leak from the ER is dependent and specific to the amino acid sequence of Aβ_42_. Taken together, these results suggest that Aβ_42_ oligomers trigger a Ca^2+^ leak from the ER, which does not depend upon a direct interaction with InsP_3_Rs.

## Discussion

Here we show that the oligomeric form of the AD-associated peptide Aβ_42_ has potent Ca^2+^ mobilizing properties and we identify mechanisms responsible for its action. Using both intact and permeabilized cell assays to investigate the effects of extracellular and internalized Aβ_42_, respectively, we establish that Ca^2+^ release from the ER makes the greatest contribution to the Ca^2+^ mobilizing effects of Aβ_42_. The InsP_3_ signaling pathway also contributes to the Ca^2+^ mobilizing properties of oligomeric Aβ_42_ in intact cells. InsP_3_Rs were not required for Aβ_42_-stimulated Ca^2+^ flux in permeabilized cells ruling out a direct regulation of InsP_3_Rs by Aβ_42_.

Central to the Ca^2+^ hypothesis of amyloid toxicity is the property of Aβ to induce Ca^2+^ elevations in its target cells. This sets in motion a cascade of events, which culminates in neuronal death. Ever since this hypothesis was put forward more than 20 years ago (Khachaturian, 1989, 1994), numerous reports have described Aβ-induced changes in intracellular Ca^2+^ in a number of cell types including primary neurons and astrocytes as well as neuroblastoma cell lines (Abramov et al., 2004b; Demuro et al., 2005). While there is general consensus that Aβ affects Ca^2+^ homeostasis, the mechanisms underlying this action of Aβ are many. Contributing to this diversity are the different experimental models used, the peptide sequence applied, the conformational state of the peptide and the method used for peptide preparation. Indeed, a number of shorter Aβ sequences have been employed in *in vitro* studies and depletion of ER Ca^2+^ store content reported (Ferreiro et al., 2004, 2008). Since Aβ_42_ is considered to be more relevant to the pathology of AD, we focused on its effects on intracellular Ca^2+^ homeostasis. Not only is an accumulation of Aβ_42_ observed in AD, this longer and more hydrophobic peptide is also more prone to self-assemble than Aβ_40_, the other principle length at which Aβ occurs. As a result, Aβ_42_ exerts a greater degree of neurotoxicity (Jarrett and Lansbury, 1993). Consistent with the growing body of evidence that soluble oligomeric forms of Aβ constitute the primary neurotoxic species (Walsh et al., 2002; Gong et al., 2003; Cleary et al., 2005; Klyubin et al., 2005), this species of Aβ_42_ potently induced Ca^2+^ fluxes and cytotoxicity in this study (Figures 1, 2 and Figure S2). Highlighting the requirement for appropriate peptide controls when studying Aβ_42_, Ca^2+^ release and cytotoxicity was also induced by a scrambled peptide sequence of Aβ_42_, although the magnitude of these responses was significantly lower than that induced by the wild type sequence. From these results, we concluded that the peptide sequence of Aβ_42_ was the major contributor to the toxicity and Ca^2+^ mobilizing properties. The temporal properties of the Ca^2+^ transients we observed were reminiscent of those reported elsewhere, being relatively slow in reaching peak and returning to baseline levels after a few minutes (Demuro et al., 2005; Simakova and Arispe, 2006). The return of these Ca^2+^ signals to baseline does, however, suggest that the Ca^2+^ elevations induced by Aβ_42_ were not immediately toxic. The Ca^2+^ mobilizing properties of the scrambled peptide, however, may reflect the previously described intrinsic properties of an oligomeric/amyloid peptide (Bucciantini et al., 2002; Yoshiike et al., 2008). For example, oligomeric forms of polyQ and insulin have been shown to induce Ca^2+^ transients (Demuro et al., 2005). The solvent HFIP used for preparation of the peptide has also previously been shown to exhibit cytotoxicity and to affect ion conductance of membranes (Capone et al., 2009).

Both Ca^2+^ influx from the extracellular space and release from ER-localized intracellular stores have been reported to be induced by Aβ and involved in its toxic action (Blanchard et al., 2004; Ferreiro et al., 2004, 2006; Kayed et al., 2004; Demuro et al., 2005, 2011; Kelly and Ferreira, 2006; Simakova and Arispe, 2006; Arispe et al., 2007; De Felice et al., 2007; Resende et al., 2008; Demuro and Parker, 2013). Although Ca^2+^ entry from the extracellular space was a component of the Ca^2+^ elevation induced by Aβ_42_ in this study, the greatest contribution was due to release from the ER. Moreover the lack of an effect of removal of extracellular Ca^2+^ upon the initial peak of the Ca^2+^ response or the number of responding cells suggested that Ca^2+^ entry across the plasma membrane was secondary to Ca^2+^ release from the ER. Since Aβ_42_ was acting to deplete the ER stores, the Ca^2+^ influx could arise via a store-operated Ca^2+^ entry pathway. These observations are not, however, incompatible with an additional mechanism for Ca^2+^ entry via plasma membrane pores formed by Aβ_42,_ which have been shown to require a longer period to develop (Demuro et al., 2011). Whether the Ca^2+^ fluxes associated with the formation of membrane pores, which were generally local to the pore and were of a relatively small magnitude, contribute to the global Ca^2+^ transient is not clear (Demuro et al., 2011).

Analysis of the mechanisms underlying Ca^2+^ release from the ER revealed that while InsP_3_Rs contributed to Aβ_42_-induced Ca^2+^ release from the ER in intact cells, the greater part of the Ca^2+^ elevation induced by Aβ_42_ was due to an alternative mechanism. However, IICR did not contribute to the Ca^2+^ responses induced by scrambled peptide. From these results, we concluded that Aβ_42_-induced Ca^2+^ release from the ER comprises an Aβ_42_ sequence-specific component, which is InsP_3_-dependent, and a second component, which is peptide sequence- and InsP_3_-independent. Comparison of these Aβ_42_ and Aβ_42_ scrambled datasets reveals that although the InsP_3_-dependent component of the total Aβ_42_ signal is relatively minor, when considered as a fraction of the Aβ_42_-specific Ca^2+^ signal (i.e., Aβ_42_—Aβ_42_ scrambled Ca^2+^ transient), its importance is increased.

Our demonstration of the participation of InsP_3_ signaling in Aβ_42_-induced Ca^2+^ responses provides robust evidence in support of this pathway in Aβ_42_-mediated Ca^2+^ signals thus far. In particular, the use of InsP_3_ 5′phosphatase overexpression to suppress InsP_3_ signaling is a highly selective strategy, overcoming issues regarding incomplete knockdown of InsP_3_Rs and contribution of the isoforms not targeted when using siRNA approaches. The inhibition of Ca^2+^ signals by caffeine is also consistent with a role for the InsP_3_ signaling pathway in the Ca^2+^ mobilizing effects of Aβ (Parker and Ivorra, 1991; Bezprozvanny et al., 1994). Not only does caffeine inhibit InsP_3_Rs directly (Bezprozvanny et al., 1994), by also inhibiting PLC, caffeine is a potent inhibitor of InsP_3_ generation (Taylor and Broad, 1998). These findings are consistent with the reduction in the Aβ_42_-induced Ca^2+^ transient observed following application of the PLC inhibitor U73122 (Resende et al., 2008) although U73122 has numerous non-specific effects. The mechanism by which InsP_3_ signaling is engaged by Aβ_42_ in this study remains to be established. Since the effects of inhibition of InsP_3_ signaling persist in the absence of extracellular Ca^2+^, activation of PLC and InsP_3_ generation by Aβ_42_-stimulated Ca^2+^ influx can be excluded. Thus, a more likely scenario would involve Aβ_42_ engagement of a PLC-coupled G protein coupled-receptor (GPCR). Indeed, a number of different GPCRs, including metabotropic glutamate receptors, are activated by Aβ_42_, contributing to modulation of LTP, Aβ_42_ synthesis and processing and cytotoxicity (Wang et al., 2004; Thathiah and De Strooper, 2011).

The internalization of Aβ from the extracellular space (Bucciantini et al., 2004; Pierrot et al., 2004; Wirths et al., 2004; Kaminski Schierle et al., 2011) raises a further possibility that Aβ acts to either directly activate/sensitize InsP_3_Rs or to alter InsP_3_ generation/metabolism. Since significant intracellular Aβ_42_ accumulation would require up to 1 h (Bucciantini et al., 2004; Kaminski Schierle et al., 2011), it is unlikely that this endocytosed Aβ_42_ contributes to the acute modulation of Ca^2+^ fluxes observed in this study and elsewhere in intact cells. Endocytosis of Aβ_42_ may, however, contribute to the more chronic effects on Ca^2+^ homeostasis as well as cytotoxicity previously reported (Ferreiro et al., 2004, 2006; Resende et al., 2008). The possibility that Aβ_42_ could directly affect ER Ca^2+^ homeostasis from an intracellular location was therefore also considered. Using a permeabilized cell assay to allow control of cytosolic conditions and access of Aβ_42_ to the ER, an Aβ_42_-stimulated Ca^2+^ efflux from the ER was observed. Unlike that observed for intact cells, the difference between Aβ_42_ and Aβ_42_ scrambled was dramatic, revealing a highly specific effect of Aβ_42_ upon ER Ca^2+^ mobilization. These effects were observed in the absence of exogenous InsP_3_ suggesting that the effects were InsP_3_R-independent. The extensive dilution of cytosol following permeabilization of the DT40 cells would also likely preclude a contribution of Aβ_42_-stimulated InsP_3_ generation. More significantly, InsP_3_Rs were not required for the Ca^2+^ mobilizing properties of Aβ_42_, since deficiency in all three InsP_3_R isoforms did not affect the Ca^2+^ mobilizing properties of Aβ_42_. The absence of a requirement for InsP_3_Rs for Aβ_42_-stimulated Ca^2+^ flux in the permeabilized cell system does not rule out the possibility that IICR contributes to Ca^2+^ fluxes and toxicity mediated by intracellular Aβ_42_. Indeed, by activating Ca^2+^-sensitive PLC and generation of InsP_3_, Ca^2+^ mobilized by Aβ_42_ could promote IICR. Consistent with this notion, microinjected Aβ_42_ was recently shown to promote Ca^2+^ signals in Xenopus oocytes in a manner that involved InsP_3_ generation (Demuro and Parker, 2013).

The depletion of the ER Ca^2+^ store by Aβ_42_ has important implications for the mechanisms of its toxicity. Depletion of ER Ca^2+^ stores results in the accumulation of unfolded proteins and activation of the ER stress response, which via caspase 12 activation and Bap31 cleavage can subsequently induce mitochondrial apoptotic cascades (Verkhratsky, 2005; Xu et al., 2005; Mekahli et al., 2011). The engagement of InsP_3_Rs during Aβ_42_-stimulated depletion of ER Ca^2+^ may be of greater consequence. Specifically, InsP_3_R-induced Ca^2+^ release from the ER and its subsequent sequestration by neighboring mitochondria could lead to mitochondrial Ca^2+^ overload, permeability transition and death (Csordas et al., 2006). These pathways also lead to increased reactive oxygen species generation, which is commonly observed in AD (Ferreiro et al., 2004, 2008; Arduino et al., 2009; Clark et al., 2010).

The use of SH-SY5Y neuroblastoma cell line and permeabilized DT40 B-lymphocytes in this study, rather than primary neurons allowed careful dissection of the role of ER Ca^2+^ signaling to Aβ-induced Ca^2+^ signals independent from Ca^2+^ fluxes that may arise in neurons as a result of electrical or synaptic activity. Moreover, using this cell line, contributions from other Aβ targets described in neurons such as NMDA receptors are excluded. Analogous to a number of other studies in electrically non-excitable primary and cultured cells including Xenopus oocytes (Demuro and Parker, 2013) astrocytes and PC12 cells (Abramov et al., 2003, 2004a; Simakova and Arispe, 2006), our data indicates that certain of the Ca^2+^ mobilizing properties of Aβ_42_ are neuron-independent and do not require the expression of any other of its reported targets. Fundamental aspects of the Ca^2+^ mobilizing properties of Aβ_42_ were further revealed and exemplified by the Aβ_42_-stimulated Ca^2+^ flux from the InsP_3_R-deficient ER of permeabilized DT40 B-lymphocytes. These latter data demonstrate for the first time that Aβ_42_ has the capacity to directly induce Ca^2+^ flux from the ER. Given the importance of the ER and InsP_3_Rs in neuronal functions, future studies will be required to test whether InsP_3_Rs contribute to Aβ-mediated neuronal pathology.

## Author contributions

Laura E. Jensen: substantial contributions to conception and design, acquisition, analysis and interpretation of data as well as writing of manuscript. H. Llewelyn Roderick: substantial contributions to conception and design, interpretation of data as well as writing of manuscript. Geert Bultynck and Tomas Luyten: designed, acquired, analysed and interpreted data of Figure 6. Hozeefa Amijee: designed, acquired and interpreted data of Figure S2. Martin D. Bootman: proof-read manuscript.

## Conflict of interest statement

Some of this work was supported by a grant from Senexis, Babraham Research Campus and Hozefa Amijee was employed by Senexis at the time this work was carried out.

## Supplementary material

Figure S1 **Validating the toxicity and conformation of Aβ_42_ oligomers. (A)** Assessment of toxicity of homogeneous Aβ preparations. Bar chart illustrating the cytotoxic effects of Aβ_42_ preparations upon SH-SY5Y cells determined using the MTT assay. Data is expressed as a percentage of MTT reduced by test samples to the dead cell controls following 24-h treatment with Aβ_42_ oligomers and scrambled Aβ at the respective concentrations. **(Bi)** Schematic diagram illustrating the epitopes of Aβ_42_ recognized by the conformation dependent, anti-oligomer antibody, A11 (Kayed et al., 2003), and the sequence dependent, anti-amyloid antibody, 12F4 (Parvathy et al., 2001). **(Bii,iii)** Sensorgrams obtained from surface plasmon resonance spectroscopy, as described (Maezawa et al., 2008). A Biacore T-100, equipped with four flow cells on a sensor chip, was used for these real-time binding studies. Biotinylated Aβ_42_was prepared by mixing a 1:10 molar ratio of biotinylated and unbiotinylated Aβ_42_. In preparation for the binding studies, Aβ_42_ was injected onto a streptavidin chip at a concentration of 10 μM to immobilize Aβ_42_by streptavidin-biotin coupling. The streptavidin chip of flow cell (Fc) 2 was partially (50%) and of Fc-4 fully saturated (100%) with Aβ_42_ oligomers. As a control, the surface of Fc-3 was partially saturated (50%) with Aβ_42_monomers. Antibodies **(Bii)** A11 and **(Biii)** 12F4 were injected over the immobilized Aβ_42_ of each flow cell at a concentration of 50 μg/ml and 10 μg/ml, respectively. The injection of the anti-oligomer antibody, A11, was followed by a regeneration step prior to injection of 12F4. The binding of injected antibodies, present in the flow phase, to the immobilized Aβ_42_was measured by response units (RU) elicited. All values were corrected for the RU obtained from the reference cell, flow cell 1, which was saturated with biotinylated Aβ_42_ only.

Figure S2 **Comparison of Ca^2+^ responses elicited by Aβ_42_ oligomers and monomers in SH-SY5Y cells. (A)** Imaging protocol employed to assess the effects of homogeneous preparations of Aβ_42_ on the Ca^2+^ signaling capacity of fluo-4-loaded SH-SY5Y cells. Cellular Ca^2+^ responses were recorded by wide-field epifluorescence. The magnitude of Ca^2+^ responses elicited by 5 μM Aβ_42_ monomers and oligomers and the subsequent application of 100 μM CCH is presented as **(B)** percentage of responding cells, **(C)** peak amplitude and **(D)** integral of the response. Soluble Aβ monomers and Aβ oligomers were prepared as previously described (Demuro et al., 2005). This method of Aβ preparation reportedly results in homogeneous populations of Aβ monomers and oligomers (also Figure S1B). All Aβ_42_ concentrations stated were based on the molar mass of the peptide.

Figure S3 **Human neuroblastoma SH-SY5Y cells lack RyR expression. (A)** Representative Ca^2+^ trace illustrating that SH-SY5Y cells do not elicit Ca^2+^ responses following the application of 10 mM caffeine, indicating that cells lack RyRs (*n* = 239 cells). However, SH-SY5Y cells do exhibit InsP_3_-mediated Ca^2+^ responses. **(B)** Immunoblot analysis corroborating the lack of RyR2 expression in SH-SY5Y cells. RyR2 expression is observed in control samples of adult hippocampal tissue and primary hippocampal cultures maintained for 4, 8, 11, and 15 days *in vitro* (DIVs).
