# Supplementary figures and images for: Alzheimer's disease-associated peptide Aβ42 mobilizes ER Ca2+ via InsP3R-dependent and -independent mechanisms
Source: Front Mol Neurosci. 2013 Nov 5;6:36. doi: 10.3389/fnmol.2013.00036 (PMC3817845; doi:10.3389/fnmol.2013.00036)

(A)

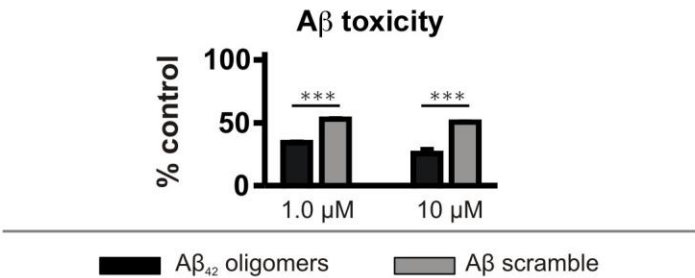

(B) *i*

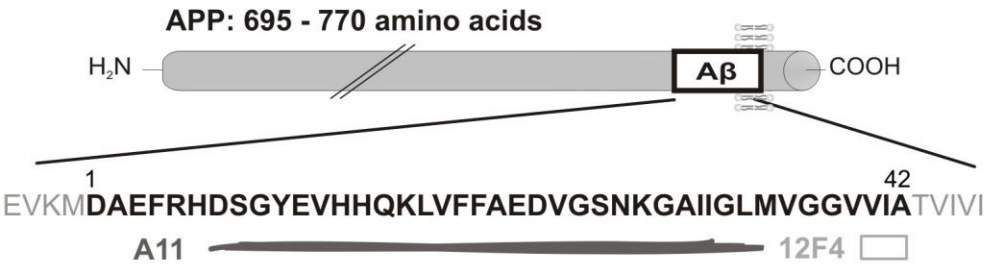

*ii*

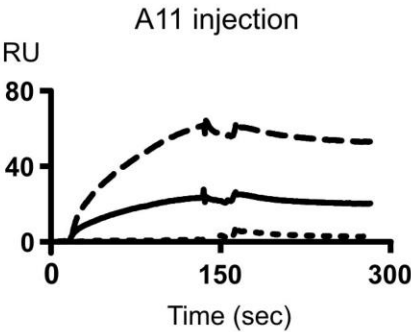

*iii*

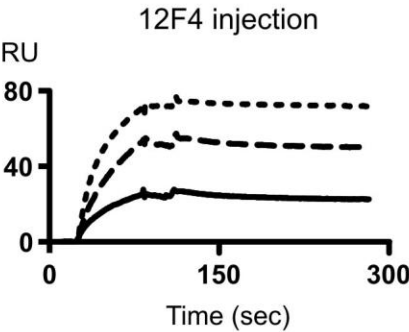

— Fc-2 A $\beta_{42}$  oligomers (50%)    - - - Fc-3 A $\beta_{42}$  oligomers (100%)  
- - - Fc-4 A $\beta_{42}$  monomers (50%)

Supplement: Figure S1 — Validating the toxicity and conformation of Aβ42 oligomers. (A) Assessment of toxicity of homogeneous Aβ preparations. Bar chart illustrating the cytotoxic effects of Aβ42 preparations upon SH-SY5Y cells determined using the MTT assay. Data is expressed as a percentage of MTT reduced by test samples to the dead cell controls following 24-h treatment with Aβ42 oligomers and scrambled Aβ at the respective concentrations. (Bi) Schematic diagram illustrating the epitopes of Aβ42 recognized by the conformation dependent, anti-oligomer antibody, A11 (Kayed et al., 2003), and the sequence dependent, anti-amyloid antibody, 12F4 (Parvathy et al., 2001). (Bii,iii) Sensorgrams obtained from surface plasmon resonance spectroscopy, as described (Maezawa et al., 2008). A Biacore T-100, equipped with four flow cells on a sensor chip, was used for these real-time binding studies. Biotinylated Aβ42was prepared by mixing a 1:10 molar ratio of biotinylated and unbiotinylated Aβ42. In preparation for the binding studies, Aβ42 was injected onto a streptavidin chip at a concentration of 10 μM to immobilize Aβ42by streptavidin-biotin coupling. The streptavidin chip of flow cell (Fc) 2 was partially (50%) and of Fc-4 fully saturated (100%) with Aβ42 oligomers. As a control, the surface of Fc-3 was partially saturated (50%) with Aβ42monomers. Antibodies (Bii) A11 and (Biii) 12F4 were injected over the immobilized Aβ42 of each flow cell at a concentration of 50 μg/ml and 10 μg/ml, respectively. The injection of the anti-oligomer antibody, A11, was followed by a regeneration step prior to injection of 12F4. The binding of injected antibodies, present in the flow phase, to the immobilized Aβ42was measured by response units (RU) elicited. All values were corrected for the RU obtained from the reference cell, flow cell 1, which was saturated with biotinylated Aβ42 only. [file Presentation1.PDF]

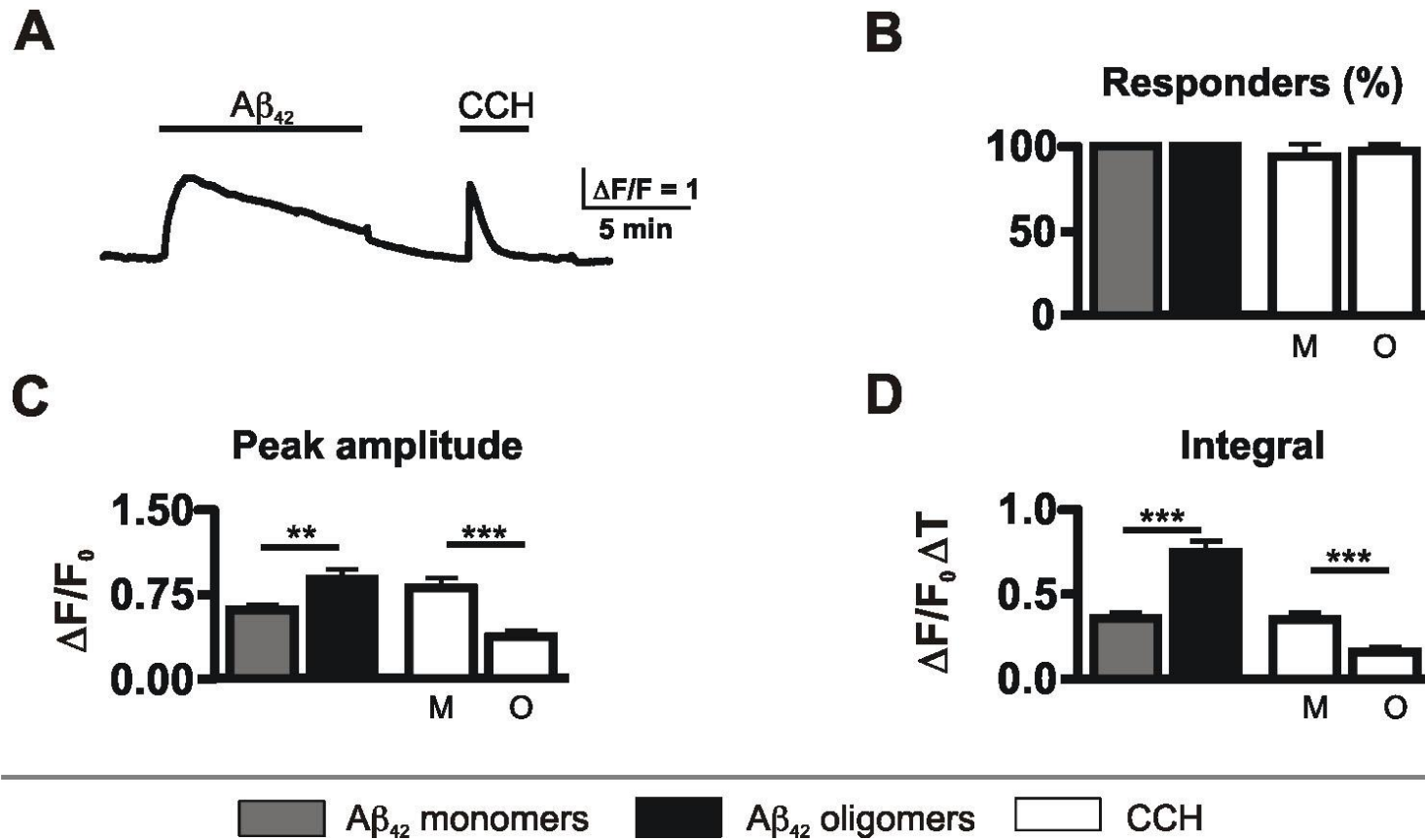

Supplement: Figure S2 — Comparison of Ca2+ responses elicited by Aβ42 oligomers and monomers in SH-SY5Y cells. (A) Imaging protocol employed to assess the effects of homogeneous preparations of Aβ42 on the Ca2+ signaling capacity of fluo-4-loaded SH-SY5Y cells. Cellular Ca2+ responses were recorded by wide-field epifluorescence. The magnitude of Ca2+ responses elicited by 5 μM Aβ42 monomers and oligomers and the subsequent application of 100 μM CCH is presented as (B) percentage of responding cells, (C) peak amplitude and (D) integral of the response. Soluble Aβ monomers and Aβ oligomers were prepared as previously described (Demuro et al., 2005). This method of Aβ preparation reportedly results in homogeneous populations of Aβ monomers and oligomers (also Figure S1B). All Aβ42 concentrations stated were based on the molar mass of the peptide. [file Presentation2.PDF]

**A**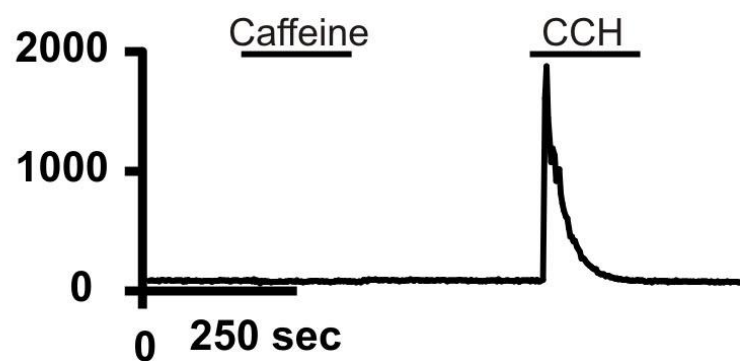**B**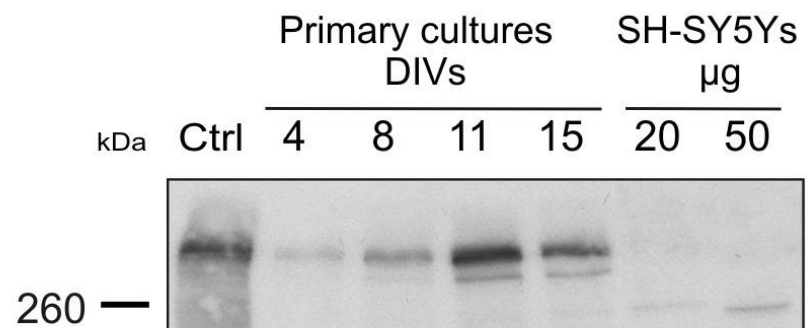

Supplement: Figure S3 — Human neuroblastoma SH-SY5Y cells lack RyR expression. (A) Representative Ca2+ trace illustrating that SH-SY5Y cells do not elicit Ca2+ responses following the application of 10 mM caffeine, indicating that cells lack RyRs (n = 239 cells). However, SH-SY5Y cells do exhibit InsP3-mediated Ca2+ responses. (B) Immunoblot analysis corroborating the lack of RyR2 expression in SH-SY5Y cells. RyR2 expression is observed in control samples of adult hippocampal tissue and primary hippocampal cultures maintained for 4, 8, 11, and 15 days in vitro (DIVs). [file Presentation3.PDF]
